# The meaning and measurement of implementation climate

**DOI:** 10.1186/1748-5908-6-78

**Published:** 2011-07-22

**Authors:** Bryan J Weiner, Charles M Belden, Dawn M Bergmire, Matthew Johnston

**Affiliations:** 1 Department of Health Policy and Management, Gillings School of Global Public Health, University of North Carolina at Chapel Hill, North Carolina, USA; 2 Cecil G. Sheps Center for Health Services Research, University of North Carolina at Chapel Hill, North Carolina, USA

## Abstract

**Background:**

Climate has a long history in organizational studies, but few theoretical models integrate the complex effects of climate during innovation implementation. In 1996, a theoretical model was proposed that organizations could develop a positive climate for implementation by making use of various policies and practices that promote organizational members' means, motives, and opportunities for innovation use. The model proposes that implementation climate--or the extent to which organizational members perceive that innovation use is expected, supported, and rewarded--is positively associated with implementation effectiveness. The implementation climate construct holds significant promise for advancing scientific knowledge about the organizational determinants of innovation implementation. However, the construct has not received sufficient scholarly attention, despite numerous citations in the scientific literature. In this article, we clarify the meaning of implementation climate, discuss several measurement issues, and propose guidelines for empirical study.

**Discussion:**

Implementation climate differs from constructs such as organizational climate, culture, or context in two important respects: first, it has a strategic focus (implementation), and second, it is innovation-specific. Measuring implementation climate is challenging because the construct operates at the organizational level, but requires the collection of multi-dimensional perceptual data from many expected innovation users within an organization. In order to avoid problems with construct validity, assessments of within-group agreement of implementation climate measures must be carefully considered. Implementation climate implies a high degree of within-group agreement in climate perceptions. However, researchers might find it useful to distinguish implementation climate level (the average of implementation climate perceptions) from implementation climate strength (the variability of implementation climate perceptions). It is important to recognize that the implementation climate construct applies most readily to innovations that require collective, coordinated behavior change by many organizational members both for successful implementation and for realization of anticipated benefits. For innovations that do not possess these attributes, individual-level theories of behavior change could be more useful in explaining implementation effectiveness.

**Summary:**

This construct has considerable value in implementation science, however, further debate and development is necessary to refine and distinguish the construct for empirical use.

## Background

Katherine Klein and Joann Sorra's [1] theory of innovation implementation has become increasingly prominent in the field of implementation science. The article in which the theory first appeared has been cited 258 times since its publication in 1996. Reflecting the theory's popularity in health and human services research, one-third of the 258 citing articles focus on innovation implementation in hospitals, physician practices, community health centers, substance abuse organizations, mental health agencies, and child welfare organizations. The theory's appeal derives partly from its simplicity. Klein and Sorra [1] identified two key determinants of effective implementation: implementation climate, or the extent to which intended users perceive that innovation use is expected, supported, and rewarded; and innovation-values fit, or the extent to which intended users perceive that innovation use is consistent with their values. Although innovation-values fit seems to have garnered more attention, especially among mental health and substance abuse researchers [2-9], implementation climate is arguably the more important construct, both in terms of its role in Klein and Sorra's [1] theory and for its potential to bring theoretical and empirical coherence to the growing body of research on organizational 'facilitators and barriers' of effective implementation.

Klein and Sorra [1] developed the implementation climate construct based on an extensive review of the determinants of effective information technology implementation. They observed that organizations use a wide variety of policies and practices to promote innovation use. Examples include training, technical support, incentives, persuasive communication, end-user participation in decision making, workflow changes, workload changes, alterations in staffing levels, alterations in staffing mix, new reporting requirements, new authority relationships, implementation monitoring, and enforcement procedures. Not only do organizations vary in their use of specific 'implementation policies and practices,' but the effectiveness of these policies and practices varies from organization to organization and innovation to innovation. In some contexts, for example, the provision of high-quality training is crucial for implementation success. In other contexts, the provision of highly valued rewards, not training, makes the difference. In light of such diversity in organizational practice and variability in effectiveness, Klein and Sorra [1] developed the construct of implementation climate to shift attention to the collective influence of the multiple policies and practices that organizations employ to promote innovation use. Implementation climate is a shared perception among intended users of an innovation, of the extent to which an organization's implementation policies and practices encourage, cultivate, and reward innovation use. The stronger the implementation climate, they assert, the more consistent high-quality innovation use will be in an organization, provided the innovation fits intended users' values. Moreover, if implementation climates of equal strength can result from different combinations of implementation policies and practices, as Klein and Sorra [1] claim, then a focus on implementation climate could bring theoretical parsimony and greater cumulativeness to scientific knowledge about the organizational determinants of innovation implementation.

Despite the construct's potential value to the field of implementation science, several conceptual and methodological problems threaten to undermine its theoretical distinctiveness and empirical utility. First, the construct has suffered from theoretical neglect. Less than a third of the 258 articles citing Klein and Sorra's [1] work discuss implementation climate, and many that do refer to the construct do so only in passing. Second, researchers have sometimes treated implementation climate as synonymous with related, yet distinct constructs such as receptive organizational context [10,11], supportive organizational context [12], and organizational culture [13]. Third, notwithstanding the widespread appeal of Klein and Sorra's [1] theory, the construct of implementation climate has been assessed empirically in only six studies [14-19], one of which was qualitative assessment [15]. Regrettably, three of the five quantitative studies exhibit levels of analysis problems (*i.e*., the statistical models were mis-specified), a flaw that raises concerns about the interpretation and value of the research findings. Finally, and not surprisingly, given the dearth of empirical research just noted, no standard instrument exists for measuring implementation climate. Few instruments have been used more than once, each instrument differs somewhat in content, and none has been systematically assessed for reliability and validity at the appropriate (organizational) level of analysis.

In this article, we clarify the meaning of implementation climate and distinguish it from other constructs important in implementation science. In addition to exploring conceptual matters, we discuss the levels of analysis issue and other measurement considerations upon which the proper testing of the theory and the utility of the construct in implementation research depend. Our intent in exploring these conceptual and methodological concerns is to promote further scholarly discussion of this important construct and foster the cumulative production of knowledge about the organizational determinants of effective implementation.

## Discussion

### What is implementation climate?

Klein and Sorra [1, p. 1060] define implementation climate as 'targeted employees' shared summary perceptions of the extent to which their use of a specific innovation is rewarded, supported, and expected within an organization.' Six features of this definition have important conceptual and methodological implications.

First, and most importantly from a conceptual standpoint, implementation climate has a specific strategic focus: innovation implementation. Unlike organizational climate, culture, or context, implementation climate does not describe a general state of affairs in an organization. As early as 1975, Schneider [20] recognized that climate, as an abstract construct, seems to include organizational members' perceptions of anything and everything that occurs in an organization. Giving the construct a strategic focus narrows attention to organizational members' perceptions of those organizational policies, practices, and procedures that promote a specific behavior or outcome (*e.g.*, innovation implementation). This not only sharpens the construct's conceptual boundaries, Schneider argues [20,21], it also increases the construct's predictive validity by emphasizing perceptions that are psychologically proximal to the behavior or outcome of interest (*e.g.*, implementation). Since Schneider's critique [20], scholars have proposed, theorized, and assessed climates for service [22-25], safety [26,26-33], creativity [34-38], and justice [39-43]. Although disparate in their strategic focus, these climates 'for something,' like implementation climate, focus on organizational members' shared perceptions of policies, practices, and procedures that orient behavior toward a specific organizational goal.

Second, implementation climate not only focuses on innovation implementation, but is also innovation-specific. Following Schneider [20], Klein and Sorra [1] insist that multiple implementation climates can exist simultaneously in an organization. Thus, a strong implementation climate can exist for one innovation (*e.g.*, clinical decision support) and not another (*e.g.*, patient-centered medical homes) if organizational members perceive differences in the extent to which innovation use is expected, supported, and rewarded. Although conceptually distinct, implementation climates for different innovations could be empirically correlated if the same implementation policies and practices pertain to multiple innovations, or the broader organizational climate, culture, or context that exists in the organization exerts a strong and pervasive influence on organizational members' perceptions and actions.

Third, Klein and Sorra [1] use the term 'targeted employees' to refer to those organizational members who are expected either to use an innovation directly (*e.g.*, front-line staff) or to support an innovation's use (*e.g.*, information technology specialists, supervisors). We use the term 'organizational members' rather than targeted employees because, in healthcare, the expected users of an innovation are not always employed by the implementing organization (*e.g.*, private-practice physicians with hospital privileges). As we discuss later, the idea that implementation climate embraces the perceptions of both expected innovation users and innovation supporters has implications for sampling and measurement.

Fourth, implementation climate refers to organizational members' shared perceptions, not to their individual or idiosyncratic views. Climate researchers have long recognized that climate is a multilevel construct [20,21,44-51]. It can be conceived and assessed at the organizational, unit, group, or individual level of analysis. Klein and Sorra [1] construe implementation climate as an organization-level construct and focus on organizational members' shared perceptions because innovation implementation in organizations is often a collective endeavor, with many people contributing something to the implementation effort. Electronic health records, chronic care models, open access scheduling, patient-centered medical homes, rapid response teams, quality improvement programs, and patient safety systems are examples of innovations that exhibit implementation complexity (*i.e.*, implementation tasks must be coordinated across people, departments, shifts, or locations) and outcome interdependence (*i.e.*, anticipated benefits depend on collective, not just personal, innovation use). For such innovations, implementation problems are likely to arise if some expected users and supporters perceive that innovation use is expected, supported, and rewarded, while others do not. We discuss this point further in a later section.

Fifth, implementation climate refers to organizational members' 'summary' perceptions of the extent to which the innovation use is expected, supported, and rewarded. Similar to other climate researchers [20,22,47,50,52], Klein and Sorra see implementation climate as a gestalt perception of the multiple and various policies and practices that an organization puts into place to promote innovation use. The focus on gestalt perceptions is consistent with their view that implementation policies and practices are cumulative, compensatory, and equifinal. Generally speaking, the more implementation policies and practices the organization uses, the better; however, the presence of some high-quality policies and practices could compensate for the absence, or low quality, of other policies and practices. For example, high-quality in-person training could substitute for poor-quality program manuals. Finally, as suggested earlier, different mixes of policies and practices can produce equivalent implementation climates. This implies that implementation climate should be measured as a composite of organizational members' perceptions of implementation policies and practices.

Finally, implementation climate focuses on organizational members' perceptions, not their attitudes. Like other climate researchers [17,49,53], Klein and Sorra [1] emphasize that climate perceptions are descriptive, not evaluative, in content. This means that implementation climate is not synonymous with organizational members' satisfaction with or appraisal of the innovation itself (*e.g.*, perceived need, level of evidence) or the organization's implementation policies and practices (*e.g.*, satisfaction with training or technical assistance). We discuss the measurement implications of this point in a later section.

### What generates implementation climate?

**Figure 1:**
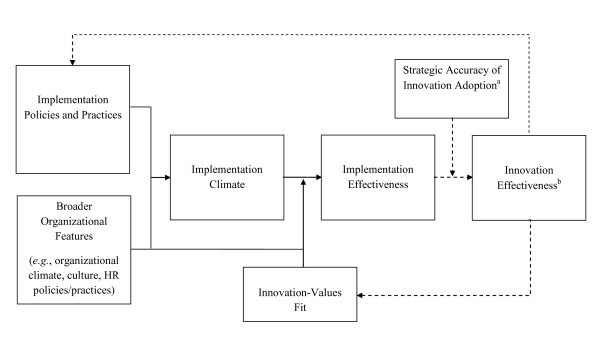
**Implementation climate: its antecedents, consequences, and modifiers**. Dashed lines indicate relationships discussed by Klein and Sorra (1996), but not discussed in this article. a. Strategic accuracy of innovation adoption (not discussed in this article) refers to the innovation's 'fit' with the strategic problem its adoption is intended to solve. b. Innovation effectiveness (not discussed in this article) refers to the benefits an organization receives as a result of its implementation of a given innovation.

Organizations can create a positive climate for implementation by employing a variety of policies and practices to enhance organizational members' means, motives, and opportunity for innovation use (see Figure 1). For example, organizations can create a positive climate by making sure that expected innovation users have easy access to high-quality training, technical assistance, and documentation (all of which enhance knowledge and skills); engaging expected users and supporters in decision making about innovation design and implementation, providing incentives for innovation use, and providing feedback on innovation use (all of which enhance motivation), and by making the innovation easily accessible or easy to use, giving expected users time to learn how to use the innovation, and redesigning work processes to fit innovation use (all of which increase opportunities or remove obstacles). Klein and Sorra use the shorthand phrase 'implementation policies and practices' to refer to the array of strategies that organizations put into place to promote innovation use. Implementation policies and practices can be temporary measures that intentionally or naturally disappear when the consistency and quality of innovation use reaches desired levels. Alternatively, they can remain in place long after initial or early implementation in order to support and reinforce continued innovation use.

Although implementation policies and practices are the primary basis for implementation climate perceptions, broader organizational features like organization climate, culture, or context may also play a role. Theory and research on the subject is limited. However, in their study of teachers' use of new computer technology in science education, Holahan *et al. *[16] found that organizational receptivity toward change was positively associated with implementation climate, and implementation climate fully mediated the effect of organizational receptivity toward change on teachers' innovation use. Similarly, building on his empirical work on service climate in banks [22], Schneider [21] proposed that service climate is influenced not just by specific organizational routines to promote good customer service, but also by 'deeper' organizational attributes, such as general human resource practices. More research is needed, but it may be the case that implementation climate arises from an amalgam of implementation policies and practices and broader organizational features. This amalgam is likely to be complex. An organization that values innovation and experimentation, for example, might not need to offer specific rewards or incentives for innovation use. Cultural values alone might be sufficient to support a positive implementation climate. On the other hand, an organization that values tradition and caution might find it essential to offer specific rewards or incentives for innovation use. These rewards or incentives would have to be powerful to counteract the dampening effect of the organization's culture on implementation climate.

Klein and Sorra [1] suggest several processes through which organizational members develop, or could develop, shared implementation climate perceptions. First, shared perceptions could result from organizational members' shared experiences with, observations of, and discussions about the organization's implementation policies and practices. Consistent leadership messages and actions could also promote common understandings among organizational members of the goals, tasks, roles, and performance expectations associated with innovation use [28,29,54-56]. Finally, broader organizational processes like attraction, selection, socialization, and attrition might also play a role [17,57,58]. By increasing the similarity in organizational members' backgrounds, experiences, values, and beliefs, these broader organizational processes increase the likelihood that organizational members will hold similar perceptions of the organization's implementation policies and practices. Conversely, organizational members are unlikely to hold common perceptions of implementation policies and practices when intra-organizational units have limited opportunity to interact and share information, when leaders communicate inconsistent messages or act in inconsistent ways, or when organizational members do not have similar backgrounds, experiences, values and beliefs.

With its emphasis on shared perception, the construct of implementation climate implies a high level of agreement in organizational members' perceptions of implementation policies and practices. The degree of 'within-group agreement' should be tested, not assumed, because, as just indicated, organizational members can vary in their perceptions of implementation policies and practices. The absence of shared perception, or put differently, the presence of high 'within-group variability,' implies that implementation climate does not exist. In other words, there is no shared meaning about the organization's implementation policies and practices [45,57].

High within-group variability, however, can be theoretically meaningful in its own right. In recent years, climate researchers have distinguished climate strength (the degree of within-group variability in perceptions) from climate level (the average magnitude of perceptions), and proposed that the former moderates the effect of the latter [24,39,54,56,59]. Building on Mischel's [60] idea of situational strength, they argue that people behave more uniformly in situations that provide clear, powerful cues about the desirability of potential behaviors. By contrast, individual differences govern behavior when situations provide ambiguous or weak cues. It follows that when implementation climate is both strong (*i.e.*, shared) and positive, organizational members are collectively more likely to use an innovation. Conversely, when implementation climate is both strong (*i.e.*, shared) and negative, they are collectively less likely to use an innovation. When implementation climate is weak (*i.e.*, not shared), organizational members are likely to vary in their innovation use as a function of individual differences (*e.g.*, personality traits, personal values) or, in complex organizations, group differences (*e.g.*, inter-unit variability in implementation climate). The moderating effect of climate strength on climate level has not been tested in implementation research, but it does receive support from studies of service climate and team climate [24,39,54,59].

### What outcomes result from positive implementation climate?

Klein and Sorra [1, p. 1058] propose that implementation climate is positively associated with implementation effectiveness, which they define as 'the overall, pooled or aggregate consistency and quality of [organizational members'] innovation use.' Like implementation climate, these authors conceive implementation effectiveness as an organization-level construct. Although they recognize that individuals and groups can vary in their innovation use, they emphasize organizational members' pooled or aggregate innovation use. This emphasis is consistent with their theoretical focus on innovations that require active, coordinated use by many organizational members (*e.g.*, electronic health records). For such innovations, they argue, implementation is more effective--and more likely to generate anticipated benefits--when all expected users use the innovation consistently and well than when some expected users use the innovation consistently and well while others use it inconsistently or poorly.

Few studies have quantitatively tested Klein and Sorra's [1] theory of innovation implementation in organizations. However, there is some evidence to support their prediction that implementation climate is positively associated with implementation effectiveness. For example, Holahan *et al. *[16] found that implementation climate was positively associated with both the quality and consistency of teachers' use of new computer technologies in science education in 69 K-12 schools in New Jersey. Klein *et al. *[61] found that the implementation climate was positively associated with consistent, high-quality use of advanced computerized manufacturing technology in 39 plants located across the United States. However, Klein *et al. *measured implementation climate as the extent to which innovation implementation was perceived to be important (or a priority) in the organization. This slippage between the construct's conceptual and operational definitions renders the meaning of the study's findings ambiguous. Consistent with Klein and Sorra's [1] predictions, Dong *et al. *[14] found in their study of large-scale information systems implementation that implementation effectiveness was highest when implementation climate was positive and innovation-values fit was present. Likewise, Osei-Bryson *et al. *[18] found in their study of enterprise resource planning systems that implementation climate was significantly associated with implementation effectiveness. It is important to note that the latter two studies measured and analyzed implementation climate at the individual level of analysis rather than the organizational level of analysis at which the implementation construct is formulated. Caution should be exercised in attributing their study results to the organizational level of Klein and Sorra's [1] theory. Doing so could result in drawing erroneous conclusions or, in the language of multi-level organizational research, committing a fallacy of the wrong level [57,62-65].

### What is the appropriate level of analysis for implementation climate?

Levels issues arise when incongruence occurs between or among the level of theory, the level of measurement, or the level of statistical analysis [45,57,64]. Implementation climate is one of many constructs that are potentially relevant to implementation science that can be conceptualized at an organizational level of theory even though the source of data for the construct resides at the individual level (*i.e.*, the level of measurement). Other constructs that fit this description include leadership, culture, power, participation, and communication.

In proposing constructs where the level of theory and the level of measurement do not match, researchers should specify the composition model or functional relationship that links the lower-level data to the higher-level construct [45,57,64,66,67]. Several composition models exist [67]. In the case of implementation climate, Klein and Sorra [1] propose a functional relationship of homogeneity--that is, they posit that organizational members share sufficiently similar perceptions of implementation climate that they can be characterized as a whole. Because both implementation climate and implementation effectiveness are formulated as organization-level constructs, an appropriate test of the relationship between these constructs should take place at the organizational level of analysis. Before proceeding with such an analysis, however, it is important to verify that the data conform to the level of the theory--that is, that the functional relationship specified in the composition model holds for the data in question [57,64]. This means ensuring that sufficient within-group agreement exists to justify aggregating individuals' implementation climate perceptions to the organizational level of analysis.

Implementation scientists can use several measures to verify that sufficient within-group agreement exists, including r_wg_, eta-squared and two intraclass correlation coefficients, ICC(1) and ICC(2). As Klein and Kozlowski [45] note, each offers a different, yet complementary assessment. R_wg _answers the question: how high is within-group agreement on a given variable for a given unit (*e.g.*, organization)? Eta-squared and ICC(1), by comparison, answer the question: to what extent does a measure vary between-units versus within-units? ICC(2) answers the question: how reliable are the unit means within a sample? An extensive literature describes the statistical assumptions, merits, limitations, and interpretative rules of thumb for these measures [45,66,68-74]. Climate researchers often assess within-group agreement using multiple measures [17,24,25,27,28,52,61,75,76]. However, different measures can produce different results depending on the number of units, the number of respondents per unit, and the amount and distribution of missing data between and within units [68-74,77,78].

The r_wg _differs from the other three measures discussed here in that it assesses within-group variability for individual units (*e.g.*, organizations). The others compare within-group variability to between-group variability across an entire sample of units. The advantage of the r_wg _is that it allows researchers to assess the extent to which units vary in the level of within-group agreement in implementation climate perceptions. What, though, should a researcher do with those units for which the r_wg _does not exceed 0.70, the rule-of-thumb value for justifying aggregation of individual perceptions to the unit-level? Klein *et al. *argue that such units should be excluded from further analysis because the implementation climate is not present in these units: no shared meaning exists [45,57]. If the data from these units do not conform to the level of theory, including these units in a statistical analysis of between-group differences can prove misleading. Construct validity issues arise [45,57,66]. For example, if one-half of the members of a unit describe the implementation climate as positive and the other one-half describe it as negative, then the average of members' perceptions of implementation climate describes none of the members' views. One could examine whether units with higher within-group agreement in implementation climate perceptions differ from those with lower within-group agreement on outcomes such as variability in organizational members' innovation use. However, such an analysis would represent a shift in the research question under investigation.

### How should implementation climate be measured?

Implementation scientists wishing to assess implementation climate face a twofold measurement dilemma: no standard instrument exists for measuring implementation climate, and existing instruments contain items specific to information systems implementation that have questionable relevance for implementation research in health and human services (*e.g.*, access to internet resources, 'help desk' availability). Although existing instruments could be adapted, changes in item content or item wording could reduce the instruments' comparability and alter their psychometric properties. For those interested in developing implementation climate measures, five guidelines follow from the conceptual discussion above (see Appendix 1 for an example of how we are following these guidelines in a study).

First, climate researchers stress that climate measures should be descriptive in content, not evaluative, in order to distinguish climate from related constructs, like attitudes or satisfaction [17,49,53]. Survey items should ask organizational members to indicate 'whether relatively objective and neutral descriptions of the work environment are accurate or inaccurate,' rather than asking them to 'rate evaluative (positive or negative) descriptions of their work environment, in light of their own values, experiences, and expectations' [17: p. 6]. Descriptive item examples include: 'Supervisors praise employees for using [innovation] properly,' 'Employees have enough time to do their work and learn new skills associated with [innovation],' and 'Technical assistance is readily available for [innovation].' Evaluative item examples include 'I'm discouraged from using [innovation],' 'I think [innovation] is a waste of time and money for our organization,' and 'I'm satisfied with the technical assistance for [innovation].' While this advice has merit, Klein *et al. *[17] note that writing purely descriptive items is difficult because, in describing relatively positive or negative policies or practices (*e.g.*, praise, expectation, monitoring), descriptive items take an evaluative tone. They suggest that climate researchers view the descriptive-evaluative distinction as a continuum rather than a dichotomy, yet stay on the descriptive side of the continuum.

Second, theory and research suggest that the wording of survey items can influence not only the variability in a construct, but also the relationship between a construct and outcomes [17,44]. Specifically, items with group (*e.g.*, organizational) referents rather than individual referents may increase the within-group agreement and between-group variability in climate measures. Glick [49] argues that survey items that direct respondents' attention to their individual experiences (*e.g.*, 'I' or 'my') encourage them to look within and ignore the experiences of others; conversely, items that direct respondents' attention to groups or higher units (collectivities) encourage them to consider the common or shared experience of others. In their study of not-for-profit community service organizations, Baltes *et al. *[44] found that psychological climate measures that differed only in their referents (individual versus organizational) were not only empirically distinguishable from one another, but each uniquely predicted job satisfaction. Moreover, discrepancies in employees' climate perceptions measured with organizational and individual referents (*e.g.*, differences in employees' perceptions of the 'average' or 'typical' employees' experience versus their own experience) also predicted job satisfaction. The findings, and others [17], suggest that survey items that differ only in referent may in fact assess closely related but nevertheless subtly different constructs. Emphasis should be placed, therefore, on items with group (organizational) rather than individual referents.

Third, researchers should assess implementation climate with items that directly measure the extent to which innovation use is perceived to be expected, supported, and rewarded. This guideline contradicts the current practice of assessing the construct with items that measure perceptions of the availability and adequacy of various implementation policies and practices [14,16,18,19]. Current practice ignores the equifinality of implementation policies and practices. If different mixes of policies and practices can generate equivalent implementation climates, then there is little reason to expect consistent relationships between specific implementation policies and practices and implementation climate. In some organizations, for example, the availability and adequacy of supervisor praise for innovation use could serve as a good indicator (indirect measure) of implementation climate. In other organizations, say those that rely primarily on financial incentives to reward innovation use, the availability or adequacy of supervisor praise would make a poor, or even irrelevant, indicator of implementation climate. A better approach for measuring implementation climate, we suggest, is to develop items that focus directly on perceived expectations, support, and rewards for innovation use. With regard to an open-access scheduling innovation, for example, direct measures could include 'Physicians in this practice are expected to use open-access scheduling,' 'Physicians in this practice have the support they need to use open-access scheduling,' and 'Physicians in this practice are recognized for using open-access scheduling.' What is important in measuring implementation climate in this example is that physicians share the perception that innovation use is expected, supported, and rewarded; less important are the specific policies or practices that generate that perception.

Fourth, as a summary or global perception, implementation climate should be measured as a multi-item scale based on a factor analysis of items that exhibit high internal consistency. In their study of innovation implementation in manufacturing plants, for example, Klein *et al. *[61] conducted factor analyses and examined the alpha-coefficients among climate items at both the individual level and organizational level before computing an implementation climate scale and subjecting the resulting scale to within-group agreement analysis. Similarly, Holahan *et al. *[16] found that their 30 implementation climate items demonstrated high internal consistency. Although they did not run a factor analysis, they too computed a mean scale at the individual level before assessing within-group variability and aggregating teachers' climate perceptions to the school level. Neither theory nor research indicates how researchers should proceed if implementation climate items do not cohere into a single scale. Does implementation climate exist if, for example, organizational members perceive that innovation use is expected and supported, but not rewarded? If so, what are the implications of such a climate for implementation effectiveness?

Finally, Klein and Sorra [1] suggest that the 'targeted employees' whose perceptions should be assessed in measuring implementation climate include not only those expected to use an innovation directly (*e.g.*, front-line staff), but also those expected to support an innovation's use by others (*e.g.*, information technology specialists, supervisors). However, researchers conducting empirical studies, including Klein *et al. *[61], have not included the perceptions of expected supporters in their measurement of implementation climate. We also favor focusing only on the perceptions of expected users because we believe, the perceptions of expected supporters have an indirect effect, as opposed to direct effect, on innovation use. When expected supporters perceive that innovation use is not expected, supported, or rewarded, they are likely to omit or put into place poor-quality implementation policies and practices. Top managers, for example, might withhold resources. Supervisors might send mixed signals. Information technology specialists might provide lackluster technical support. In our view, the actions or non-actions of expected supporters influence innovation use by creating a favorable or unfavorable implementation climate for expected users. It is the implementation climate perceptions of expected users that are more psychologically proximal to, and therefore, like to be more predictive of, the consistency and quality of expected users' innovation use.

## Summary

Over the last decade, impressive efforts have been made to catalogue the features of innovations, organizations, and environments that influence innovation implementation [79,80]. While the volume of research on implementation is slim compared to that on adoption, the list of such factors is large and shows no signs of shrinking. These efforts to catalogue facilitators and barriers of implementation are to be applauded, especially if they stimulate the construction of testable theories to explain implementation success, or encourage the development of useful models to guide implementation processes. The challenge for building research evidence in implementation science, however, is that often, perhaps even most of the time, there are multiple ways to achieve the same outcomes. For example, there are at least three ways that organizations can create a good fit between the knowledge and skills of expected users and those demanded for consistent, high-quality use of a technically complex innovation. Organizations can raise expected users' knowledge and skills to the level required by the innovation; lower the innovation's technical complexity to match expected users' current knowledge and skills; or hire, promote, or transfer organizational members who already possess the required level of knowledge and skills. If equifinality is an essential feature of organizations, as it is of most social systems, then efforts to link specific policies and practices to implementation success are likely to produce equivocal results. Sometimes training will be associated with implementation success; sometimes it will not. Researchers could focus on identifying the conditions under which organizations use specific implementation policies and practices, such as training. Alternatively, they could focus on the cumulative impact of implementation policies and practices by examining whether positive implementation climate (regardless of how such a climate is achieved) is associated with implementation success. These options are not mutually exclusive, since they address different, and arguably important, research questions. A focus on implementation climate, however, would facilitate the comparison of implementation effectiveness across organizations that use different mixes of policies and practices to promote consistent, high-quality innovation use.

Ultimately, the value of the implementation climate construct depends on its predictive utility. We conclude, therefore, with some thoughts on how to advance empirical investigation and theoretical inquiry. First, since the construct and the theory in which it figures are pitched at the organizational level, a longitudinal multi-organizational research design provides the best means for assessing the construct's scientific worth. Although sample size and statistical power considerations make it tempting to test the theory at the intra-organizational level, caution should be exercised in using clinics, departments, or organizational divisions as units of analysis. This approach might be defensible if a reasonable case can be made that the clinics, departments, or divisions in question represent distinct (*i.e.*, independent) units of implementation. As noted earlier, though, measuring the construct and testing the theory at the intra-organizational level introduces the risk of committing the fallacy of the wrong level. Pragmatically, implementation climate might not demonstrate enough between-group variability among intra-organizational units to permit the observation of a significant association with implementation effectiveness.

Second, implementation scientists should keep in mind the type of innovation that Klein and Sorra's (1996) theory of implementation effectiveness seeks to predict and explain. Theories, like tools, have a bounded range of application. Given the theory's context of origin--the study of information systems and technology implementation in manufacturing settings--the construct of implementation climate is perhaps most useful for studying complex innovations in health and human service delivery. By complex, we mean innovations that require collective, coordinated behavior change by many organizational members in order to successfully implement them and realize some or all of the anticipated benefits of innovation use. Put differently, implementation climate is likely to prove useful in studying innovations that exhibit moderate to high levels of task interdependence and outcome interdependence. Conversely, implementation climate is not likely to prove useful in studying innovations that individual health and human service providers can adopt, implement, and use on their own with relatively modest training and support and for which they and their patients or clients can realize anticipated benefits regardless of what other providers do. For such innovations, individual or interpersonal theories of behavior change may offer more explanatory power than organization theories of innovation implementation.

Third, good measurement practice, particularly in the development of new measures, is essential for building scientific knowledge. The measurement guidelines offered above could promote consistency across studies. Yet, implementation scientists might still find it challenging to develop measures of implementation climate that are sufficiently tailored to make them predictive in specific innovation-implementation contexts, yet not so tailored that they could not be used in other innovation-implementation contexts without substantial modification. The construction of instruments that directly measure implementation climate perceptions could mitigate this tension, but it cannot eliminate it entirely. If no single instrument will meet implementation scientists' needs, then perhaps the field of self-efficacy research offers a useful model. Health behavior scientists have developed self-efficacy instruments for smoking, physical activity, and other health behaviors that are reliable and valid within their domain of application [81-88]. Although item content is tailored, the instruments are based on theory and have enough features in common that scholars can accumulate scientific knowledge across health problems.

Finally, implementation scientists should continue to develop the implementation climate construct. Several questions merit further theoretical, and empirical, attention. Is it useful, for example, to distinguish implementation climate strength from implementation climate level? Do some implementation policies and practices--or, for that matter, some broader features of organizational context--influence the strength of implementation climate but not the level of implementation climate? Likewise, are the three aspects of implementation climate (*i.e.*, expected, supported, and rewarded) equally important? Does their relative importance depend on the implementation context and, if so, how? Lastly, is implementation climate a theoretically meaningful construct at the individual level? If so, how does an individual-level analogue relate to the organization-level construct or to other important constructs in implementation science?

## Competing interests

The authors declare that they have no competing interests.

## Authors' contributions

BJW conceived the idea for the manuscript and took the lead in drafting it. MB, DB, and MJ conducted the background research that informed the manuscript, contributed ideas about the meaning of the construct, made editorial and substantive changes to manuscript drafts. All authors read and approved the final manuscript.

## Appendix 1

### Implementation climate and organizational performance in the Community Clinical Oncology Program

In a current study, we are examining the association of implementation climate, innovation values fit, and organizational performance in the Community Clinical Oncology Program (CCOP). Established in 1983, the CCOP is a three-way partnership between the NCI's Division of Cancer Prevention (NCI/DCP), selected cancer centers and clinical cooperative groups ('CCOP research bases'), and community-based networks of hospitals and physicians ('CCOP organizations') to conduct Phase III clinical trials [89,90]. In this partnership, NCI/DCP provides overall direction and funding; CCOP research bases design clinical trials; and CCOP organizations assist with patient accruals, data collection, and dissemination of study findings. As of December 2010, 47 CCOP organizations located in 28 states, the District of Columbia, and Puerto Rico participated in NCI-sponsored clinical trials. The CCOP includes 400 hospitals and more than 3,520 community physicians. In FY 2010, the CCOP budget totaled $93.6 million. The median CCOP organization award was $850,000.

CCOP organizations are led by a physician principal investigator who provides local program leadership. CCOP staff members include a program coordinator, research nurses or clinical research associates, data managers, and regulatory specialists. These staff members coordinate the selection of new clinical trial protocols for CCOP participation, disseminate protocol updates to the participating physicians, and collect and submit study data [15,90,91]. CCOP-affiliated physicians accrue or refer participants to clinical trials, and typically include medical, surgical and radiation oncologists, general surgeons, urologists, gastroenterologists, and primary care physicians. Through their membership in CCOP research bases, CCOP-affiliated physicians also participate in the development of clinical trials by proposing study ideas, providing input on study design, and, occasionally, serving as principal investigator for a clinical trial [15,90,91].

In the fall of 2011, we will survey a stratified random sample of 900 CCOP-affiliated physicians to obtain data on their perceptions of implementation climate, innovation-values fit, and other constructs. We will measure implementation climate with six items referenced to the respondent's CCOP organization:

1. Physicians are expected to enroll a certain number of patients in NCI-sponsored clinical trials.

2. Physicians are expected to help the CCOP meet its patient enrollment goals in NCI-sponsored clinical trials.

3. Physicians get the research support they need to identify potentially eligible patients for NCI-sponsored clinical trials.

4. Physicians get the research support they need to enroll patients in NCI-sponsored clinical trials (*e.g.*, consenting patients).

5. Physicians receive recognition for enrolling patients in NCI-sponsored clinical trials.

6. Physicians receive appreciation for enrolling patients in NCI-sponsored clinical trials.

Respondents will use a five-point scale to indicate whether they disagree, somewhat disagree, neither agree nor disagree, somewhat agree, or agree with each statement.

Our measurement approach is consistent with the measurement guidelines described in this paper. Specifically, the items are: descriptive versus evaluative in focus; group-referenced rather than individually referenced; direct measures of climate perceptions rather than indirect measures of specific implementation policies and practices; multiple in number for the three dimensions of implementation climate (*i.e.*, expected, supported and expected); and targeted toward respondents who are expected to use the innovation directly (*i.e.*, physicians).

Like Klein and Sorra's (1996) theory, our conceptual model emphasizes organization-level constructs. Therefore, we will conduct statistical tests to assess the extent to which responses to individual-level scales constructed from factor analysis show sufficient within-CCOP agreement to justify aggregation to the CCOP organization level. Specifically, we will compute eta-squared, ICC(1), ICC(2), and r_wg_. We will compare the values of these statistics to recommended cut-off values and values reported in other studies using individual-level variables aggregated to the organizational level [31,49]. If on balance the statistical tests justify data aggregation, we will construct CCOP-organization-level averages for implementation climate, innovation-values fit, and other organization-level constructs for which data are obtained at the individual level of measurement. Using regression analysis, we will examine the association of these variables with CCOP organizational performance, measured as number of patients enrolled in treatment trials by the CCOP organization. If the statistical tests do not justify aggregation, we will revise our hypotheses to focus on implementation climate strength and incorporate in our statistical models variables that measure intra-CCOP variability of individual responses (*e.g.*, coefficient of variation).
